# Scalable aesthetic transparent wood for energy efficient buildings

**DOI:** 10.1038/s41467-020-17513-w

**Published:** 2020-07-31

**Authors:** Ruiyu Mi, Chaoji Chen, Tobias Keplinger, Yong Pei, Shuaiming He, Dapeng Liu, Jianguo Li, Jiaqi Dai, Emily Hitz, Bao Yang, Ingo Burgert, Liangbing Hu

**Affiliations:** 1 Department of Materials Science and Engineering, University of Maryland, College Park, MD 20742 USA; 2 Center for Materials Innovation, University of Maryland, College Park, MD 20742 USA; 3 Wood Materials Science, ETH Zürich, Stefano-Franscini-Platz 3, CH-8093 Zürich, Switzerland; 4 Wood Technology, Cellulose & Wood Materials, EMPA, CH-8600 Dubendorf, Switzerland; 5 Department of Mechanical Engineering, University of Maryland, College Park, MD 20742 USA

**Keywords:** Energy, Sustainability, Materials science, Optical materials and structures

## Abstract

Nowadays, energy-saving building materials are important for reducing indoor energy consumption by enabling better thermal insulation, promoting effective sunlight harvesting and offering comfortable indoor lighting. Here, we demonstrate a novel scalable aesthetic transparent wood (called aesthetic wood hereafter) with combined aesthetic features (e.g. intact wood patterns), excellent optical properties (an average transmittance of ~ 80% and a haze of ~ 93%), good UV-blocking ability, and low thermal conductivity (0.24 W m^−1^K^−1^) based on a process of spatially selective delignification and epoxy infiltration. Moreover, the rapid fabrication process and mechanical robustness (a high longitudinal tensile strength of 91.95 MPa and toughness of 2.73 MJ m^−3^) of the aesthetic wood facilitate good scale-up capability (320 mm × 170 mm × 0.6 mm) while saving large amounts of time and energy. The aesthetic wood holds great potential in energy-efficient building applications, such as glass ceilings, rooftops, transparent decorations, and indoor panels.

## Abstract (web summary)

Transparent wood composites are promising engineered materials for green energy-efficient building. Here, authors demonstrate novel aesthetic wood with integrated functions of optical transparency, UV-blocking, thermal insulation, and mechanical strength for this sustainable application.

## Introduction

To date, the development of green, energy-saving materials has been a prevailing research topic from the perspective of sustainability in response to the rapidly growing burden of energy consumption and environmental pollution^1,2^. Natural materials such as wood and its derivatives have been regarded as one of the most important alternatives in green and energy-efficient buildings due to the abundance, renewability, low cost, and sustainability of source materials^3,4^. One latest trend in wood-based building material fabrication is the recently developed transparent wood composite, which integrates the anisotropic hierarchical wood structure with optical, mechanical, and thermal properties^5–7^. Numerous merits are endowed to the transparent wood composites including light weight, high optical transmittance, tunable haze, low thermal conductivity compared to glass and excellent mechanical robustness^8–10^. Additionally, transparent wood composites can harvest sunlight effectively due to the light guiding effect, which is meaningful for energy saving and comfortable indoor lighting. With these integrated advantages, the transparent wood composites have emerged as the promising engineering components (e.g., rooftops, windows, and transparent decorations) in green energy-efficient buildings^1,9^.

Current approaches for fabricating transparent wood composites were generally based on a complete (or nearly complete) delignification process, that is, removing most of light absorbing materials (lignin and extractives)^11–13^ or chromophoric components with lignin remaining about 80%^14^. However, the intensive chemical treatment can severely break down the original wood structure (e.g., the cell wall is partly degraded, and the growth ring patterns become less visible) to ensure efficient polymer infiltration. In addition, these previous works generally focused on the morphology and anisotropy of optical, mechanical, and thermal properties^13,15,16^, yet alternating structures, the natural aesthetics of wood’s original annual growth patterns, and scalable manufacturing via efficient process are rarely discussed.

In this work, we develop an aesthetic transparent wood (denoted as aesthetic wood) by spatial-selectively removing lignin of native wood material to make wood transparent and preserve its natural patterns simultaneously. Softwood (e.g., Douglas fir) is chosen as the proof-of-concept demonstration due to the pronounced structural contrast between its low-density earlywood (EW) and high-density latewood (LW). In a short 2 h chemical treatment, natural wood is selectively delignified to preserve its original growth ring patterns. The refractive-index-matched epoxy is then infiltrated into nanoscale framework to make the wood transparent with preserved wood patterns. Consequently, the novel concept of aesthetic wood in this work is demonstrated for the first time possessing integrated excellent functions of optical transparency, UV-blocking, thermal insulation, mechanical strength, scalability, and aesthetics. We anticipate such multifunctional aesthetic wood will hold great potential in modern green buildings.

## Results

### Fabrication of aesthetic wood

**Fig. 1:**
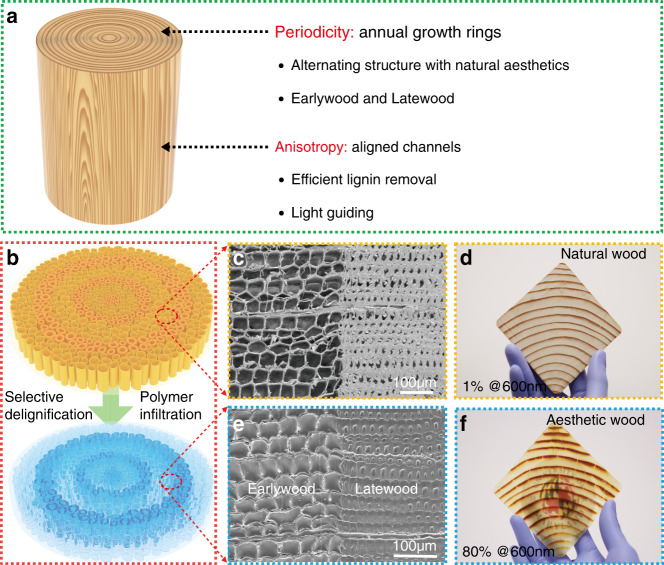
Fabrication, microstructure and appearance of aesthetic wood. **a** An indication of the design which combines the periodicity (annual growth rings) with anisotropy (aligned channels) of wood to realize a new kind of transparent wood composite. **b** Schematic to display the procedures for fabricating aesthetic wood (aesthetic wood-R) from natural wood with vertically aligned cells and annual growth rings after fast spatially selective delignification and polymer infiltration. **c**, **e** The cross-sectional SEM images of natural wood and dense aesthetic wood-R microstructures after polymer filling (there is a sharp boundary between EW and LW). **d**, **f** Photos to show a large piece of aesthetic wood-R (86 mm × 86 mm × 2 mm) with preserved wood patterns and high average transparency (80% at 600 nm) derived from Douglas fir.

We demonstrate two types of aesthetic wood based on the periodicity and anisotropy of natural wood (Fig. 1a): one type with aligned microchannels perpendicular to the wood plane is defined as aesthetic wood-R while another type with channels parallel to the wood plane is aesthetic wood-L. Natural softwood presents the intrinsic aesthetic properties of the annual growth ring patterns with alternating structures at macroscopic and microscopic scales^17^. From the macro perspective, the rings are developed by the alternating formation of EW in spring and LW in summer (Supplementary Fig. 1, softwood pine disk): the EW is usually wider, weaker, more porous and lighter in color than the LW. In their microstructure, EW cells have a relatively larger lumen diameter and thinner cell walls compared to LW^18^. By profiting from the special structural organization of Douglas fir wood, aesthetic wood not only inherited the original aesthetic from the wood itself but also possessed favorable optical, and mechanical properties. Moreover, the efficient delignification process made it possible to realize the large-scale production of transparent wood with little time and energy consumption.

Figure 1b demonstrates the fabrication process of aesthetic wood-R: natural wood is obtained by the industry-adopted cross-section cutting method with the annual growth rings visible due to the sharp microstructure difference between EW and LW (Fig. 1b–d). After spatially selective delignification using our efficient method, the EW area has become almost completely white due to the light scattering and the vast removal of light absorbers (i.e., lignin and some extractives) while the LW area preserves partial lignin. Afterwards, the refractive-index-matched polymer/epoxy was filled into the wood backbone to prepare the aesthetic wood based on this special structure. The scanning electronic microscope (SEM) image in Fig. 1e specifically reveals the maintaining dense structure after fully impregnating polymer, particularly in the LW area. Aesthetic wood-R was fabricated by this approach, which possesses not only the preserved wood patterns, but a high average transparency (80% at 600 nm, the UMD logo can be seen clearly behind the aesthetic wood-R, see Fig. 1f). This work provides new horizons and more potentials for green buildings and other construction applications, which is impossible to achieve with regular glass.

### Morphological and chemical characterizations of aesthetic wood

**Fig. 2:**
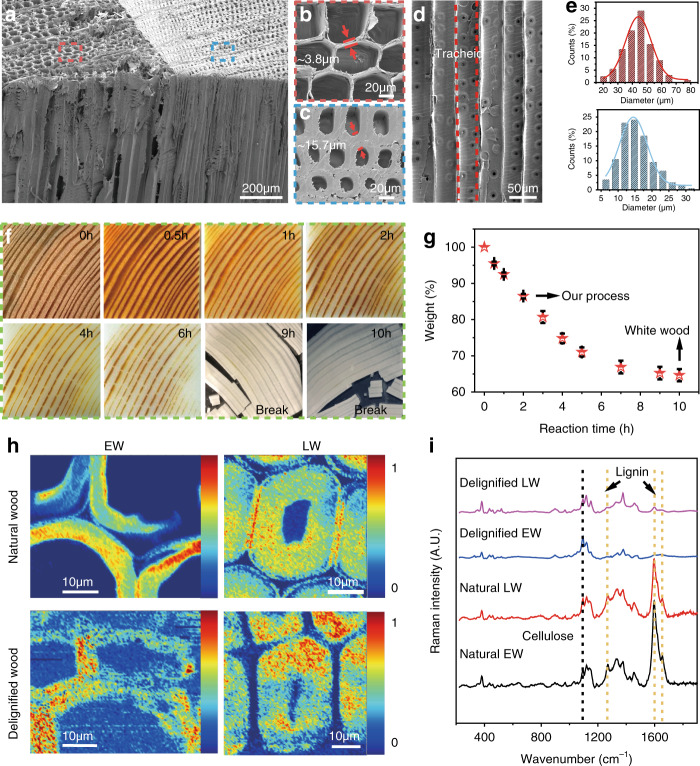
Morphological and chemical characterizations of aesthetic wood. **a** The SEM image of Douglas fir to show its mesoporous structure. **b**, **c** Magnified SEM images of EW and LW to present the differences in microstructural lumina. **d** The aligned micro-sized channels with tracheids. **e** The pore diameter distributions of EW and LW in the natural Douglas fir. **f** Photo comparison of color and pattern changes in wood templates during lignin removal process in the laboratory (0–10 h). **g** The weight loss behavior as a function of delignification process time. Error bars represent standard deviation. **h** Cell wall components of the EW and LW in natural wood (non-treated reference) and delignified wood cell wall (CW) after VCA. **i** The corresponding Raman spectra in (**h**).

Softwood (gymnosperms) generally relies on tracheids to transport water, like pine, and Douglas fir^19,20^. The structural differences between different wood species of softwood and hardwood can lead to different aesthetic results. Here, Douglas fir was chosen as a proof-of-concept demonstration, which has a pronounced contrast of both color and density between EW and LW^21^ and exhibits a unique aesthetic with obvious wood patterns. The mesoporous structure of Douglas fir is shown in Fig. 2a. There is a rather sharp boundary between EW and LW to a very indistinct separation in Douglas fir. The EW is more porous with much thinner cell walls (1.4~2.6 μm) than the LW (5~10 μm) (Fig. 2b, c). The distribution of the wood tracheids as shown in Fig. 2d (hollow tube-like structure) with a lumen diameter range of 20–80 μm and 5–35 μm involving EW and LW, respectively (Fig. 2e). As a result, the different pore-size distributions are usually indicative of different densities of EW and LW.

In a typical experiment, a wood block with dimensions of 60 mm × 60 mm × 2 mm was applied for the procedure analysis of delignification treatment. Briefly, a simple delignification process using the acidic NaClO_2_ method was employed to partial remove the colored components (mainly lignin, along with extractives) from the bulk wood. As shown in Fig. 2f, the evolution of the wood’s macroscopic color indicates the removal of the color compounds presented at the surface. Specifically, the weight loss at various points during the delignification process (0–10 h) was recorded by a balance in the dry states (dried at 100 °C for 48 h, Fig. 2g), which is mainly ascribed to the removal of lignin and a little extractive during the process. After 2 h of treatment, spatially selective delignification can be realized: the EW has become almost completely white whereas the LW kept the pattern well owing to the residual lignin and colored components. The main contributor to spatially selective delignification is the inherent structural difference between EW and LW, which accordingly leads to a faster solution diffusion in EW than in LW (Supplementary Fig. 2). The weight loss was 13.5 wt% after 2 h of treatment (Fig. 2g). After selective delignification, the nano- and macro-features of original wood were essentially preserved as well (Supplementary Fig. 3), including the wood patterns to show the nature of aesthetics. Note that it took much more time (e.g. 10 h) for LW to be completely transformed white with a corresponding weight loss of ~35 wt%. Moreover, the integrity of the delignified wood structure cannot be maintained upon long treatment times resulting in poor mechanical properties (treated 9–10 h) (Fig. 2f) owing to the distinct density difference (Supplementary Fig. 4) between the EW (284.6 kg m^−3^) and LW (846 kg m^−3^), and the complete removal of lignin, which also acts as binder among the wood cells^14,22,23^.

Note that the choice of wood species is vital to the successful fabrication of aesthetic wood. Although both hardwood and softwood are in principal suitable, hardwood possesses a significantly different structure consisting of vessels and fibers (Supplementary Fig. 5), while softwood mainly consists of tracheids^20^. Basswood, a type of hardwood, for example, has uniform cell wall thickness of around 1.3~2.9 μm (Supplementary Fig. 5c), much thinner than the cell wall thickness of LW in Douglas fir. Meanwhile, the vessel channels exhibit larger lumen diameters than narrow tracheids (Supplementary Fig. 5d), with bimodal pore-size distribution (Supplementary Fig. 5e). Consequently, owing to the almost synchronous reaction process in basswood of the EW and LW, there are almost no apparent wood patterns preserved after a couple of hours’ treatments (Supplementary Fig. 5f). The same result occurred in balsa wood (another type of hardwood), confirming that aesthetic wood with patterns is nearly impossible to fabricate from diffuse-porous hardwood possessing bimodal pores and uniform solution diffusion (Supplementary Fig. 6).

Raman spectroscopy imaging in combination with vertex component analysis (VCA), a multivariate data analysis method, were employed to assess the distribution of lignin in the wood scaffold after selective delignification^24^. The cell wall component for EW and LW in natural wood and delignified wood, respectively are shown in Fig. 2h. The corresponding Raman spectra are demonstrated in Fig. 2i, especially the characteristic bands of lignin component locate at 1598 cm^−1^, 1656 cm^−1^ and 1269 cm^−1^ (a marker band of the aryl-OH and aryl-OCH_3_ in guaiacyl (G) units in lignin)^25,26^ ascribing to aromatic C=C stretching, coniferyl alcohol C=C, C=O stretching, and C–H banding of C=C, aromatic C=C stretching, respectively. As expected, contrasting with the EW and LW in natural CWs, the representative lignin bands almost disappear in EW cell walls after delignification while they are still shown in LW cell walls. Meanwhile, respective cellulose peaks, for example at 1095 cm^−1^ (C–O–C stretching vibrations), remain unchanged after NaClO_2_ treatment^27^. These results give strong evidence to support that most of the lignin in the EW has been removed while a small proportion of the lignin in the LW remains. This phenomenon leads to the retained aesthetic wood patterns in the final aesthetic wood products.

### Scalability and the mechanical properties of aesthetic wood

**Fig. 3:**
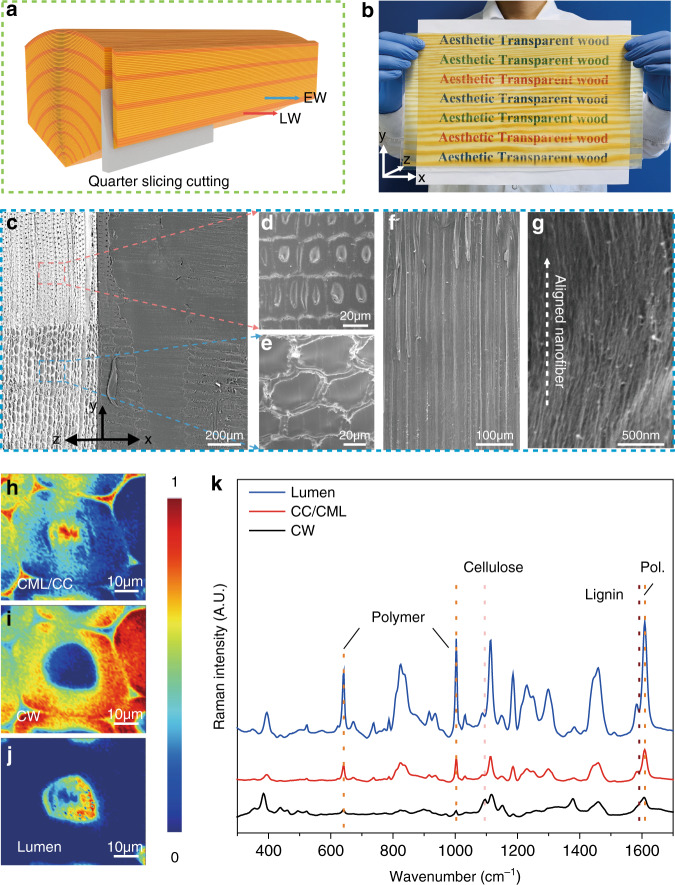
Scalability of aesthetic wood. **a** Schematic of quarter slicing cutting to obtain the wood veneer with straight-line patterns. **b** A large-scale aesthetic wood assembled by L-wood veneer (demonstrated for sample size of 320 mm × 170 mm × 0.6 mm). **c** SEM image of the preserved whole wood microstructure after filling with polymer. **d**–**e** Zoomed-in SEM images to show the EW and LW well-defined lumina full of polymer. **f**–**g** The detailed SEM image of the aligned micro-sized channels and the aligned cellulose nanofibers on the corresponding cell wall. **h**–**j** VCA of wood cells in obtained aesthetic wood. **k** Corresponding Raman spectra.

Following the same procedure, we then constructed the aesthetic wood-L with straight patterns created by the quarter slicing cutting strategy (Fig. 3a). The efficient spatially selective delignification process not only endows excellent structural integrity but also facilitates the large-scale production of aesthetic wood-L. In Fig. 3b, we demonstrate the ability to fabricate a sample size of 320 mm × 170 mm × 0.6 mm, which is significantly larger than all reported transparent woods using delignified wood as the framework (Supplementary Table 1)^1,9–11,15,16,28^. Large-scale manufacturing has been regarded as one of the major challenges for transparent wood manufacturing and industrialization. Our work points to a potential route towards addressing the manufacturing challenge of transparent wood (e.g., large scale with a short processing time). However, it is worth mentioning that thicker aesthetic wood yet without compromising its aesthetic features and other properties is preferred in order to provide better load-bearing properties in building applications, which should be considered in future research.

The straight-line patterns display the traditional symmetric aesthetic (the background is an A4 paper). In the meantime, the obtained aesthetic wood-L (with a thickness of 0.6 mm) is optically transparent (Supplementary Fig. 7), with a total transmittance of 87% and optical haze of 65% at 600 nm. To identify the compatibility between epoxy and treated wood scaffold, SEM was applied to illustrate the detailed microstructures. Figure 3c shows that aesthetic wood displays massive aligned dense microchannels along the wood growth direction after successful infiltration. Zoomed in from the top view, although the lumen of LW is much smaller than that of EW, they are all densely packed (Fig. 3d, e). Additionally, the identical channels and apertures are fully filled with polymer (Fig. 3f) which acts as a glue to create strong interaction between the cellulosic cell wall and polymer itself. Raman spectroscopy imaging was further performed to identify the distribution of the impregnating polymer in the obtained wood cell including cell corner (CC), compound middle lamella (CML) (Fig. 3h), cell wall (CW) (Fig. 3i) and lumen (Fig. 3j). According to the corresponding Raman spectrum in Fig. 3k, the strong-signal peaks within lumina indicate the bond stretching of epoxy: 640 cm^−1^ (aromatic C–H out-of-plane deformation), 1001 cm^−1^ (polyamidoamine adduct, amino groups) and 1608 cm^−1^ (aromatic ring breathing mode)^29^. Polymer signals can also be detected in the CML/CC and CW, suggesting that polymer has been well-infiltrated into the wood cells, forming robust interfaces with cellulose in the delignified wood scaffold.

From the perspective of construction materials, the mechanical properties are equally important^30^. The hierarchical cellular structure of wood leads to unique anisotropic mechanical features. As shown in Supplementary Fig. 8a, aesthetic wood-R exhibits dramatically improved tensile strength over natural R-wood (21.56 MPa vs. 6.24 MPa), while aesthetic wood-L possesses a higher tensile strength of 91.95 MPa due to the synergy between the wood matrix and filling polymer. The toughness of aesthetic wood-R and aesthetic wood-L are 0.523 MJ m^−3^ and 2.73 MJ m^−3^, respectively (Supplementary Fig. 8b), enhanced contrasting with natural wood, yet yielding the remarkably mechanical anisotropy. For energy efficient buildings, both high tensile strength and high toughness are greatly advantageous for load-bearing functions^22^. Moreover, to further reveal the details of the fracture behavior of natural wood and aesthetic wood, the fractured surface after tensile measurement of each type was characterized by SEM (Supplementary Fig. 8c–f). The porous lumen structure and aligned microchannels in the natural wood are visible after fracture. The cross-sectional SEM images of aesthetic wood-R and aesthetic wood-L after tensile tests show that the polymer is fully filled in the middle of the wood backbone and connects the separated wood fibers. The anisotropic mechanical properties of aesthetic wood can be attributed to the aligned cellulose nanofibers in the cell wall, giving rise to high strength along the fiber direction yet relatively low strength perpendicular to the tracheid direction (Fig. 3g)^8^.

### Optical properties and patterns design of aesthetic wood

**Fig. 4:**
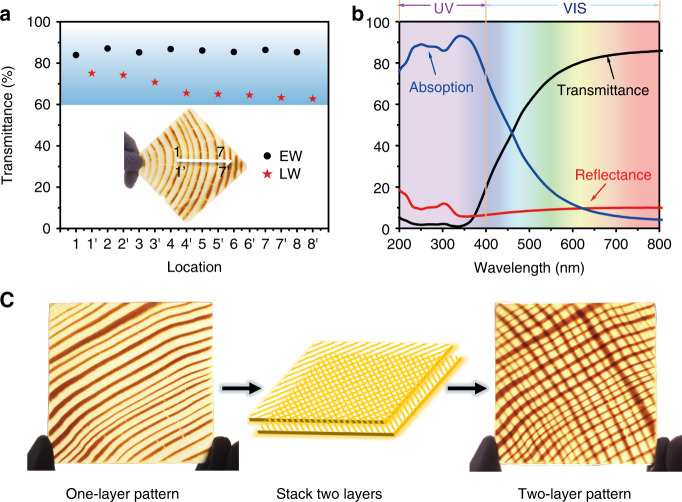
Optical properties and patterns design of aesthetic wood. **a** The transmittance in the EW and LW of obtained aesthetic wood (The locations marked 1–8 represent the EW areas while 1′–8′ represent the LW areas). **b** As-prepared aesthetic wood exhibits excellent UV-blocking performance: high absorption in 200–400 nm, high transmittance at 600 nm and low reflectance. **c** The latticed aesthetic patterns can be obtained by stacking two layers of aesthetic wood.

Previous works have demonstrated that by tuning the starting wood materials or the chemical processing parameters^31,32^, some blurred wood patterns can be maintained in the final products. It remains challenging to achieve clear and designable aesthetic patterns in transparent wood with integrated advantageous features such as a high optical transmittance, UV-blocking capability, low thermal conductivity and high mechanical strength. Our aesthetic wood, demonstrated for the first time, features a combination of these abovementioned advantages. Such combined advantages are desirable for energy efficient building applications, particularly in pattern ceiling. Owing to the inhomogeneous distribution of lignin and cell structures between EW and LW in the assembled aesthetic wood, the transmittance is not uniform. We chose 8 locations in EW and LW areas and measured the transmittance, respectively (Fig. 4a). As initially conceived, the LW area exhibits lower transmittance (Average ≈ 68%) than in EW area (Average ≈ 86%). Despite the difference of transmittance between LW and EW, the remaining pattern only slightly decreases the overall average transmittance of the aesthetic wood in the visible light region.

Furthermore, the favorable UV-blocking performance is specially expected in aesthetic wood. Solar radiation reaching the earth surface constitutes infrared radiation, visible light, and ultraviolet (UV) radiation, in which UV is made up of three bands UVA (320–400 nm), UVB (275–320 nm) and UVC (200–275 nm), the smaller wavelength has the stronger energy^33^. UVC (possessing the highest energy) is filtered by the ozone layer. UV is invisible to the human eye but can provide a hazard and damage to many materials including furniture and interior displays^34^. Particularly, 3.5% UVB and 96.5% UVA will reach the earth’s surface on a summer day. Here, the aesthetic wood with tunable UV-blocking performance over a wide range from 200 to 400 nm was fabricated successfully. The sample (2-mm thick) was treated for various treatment times. Under 2 h, the aesthetic wood was able to shield almost 100% of the UVC and UVB spectra and most of the UVA spectrum. If the reaction is prolonged to 9 h, the UVA blocking was remarkably decreased along with an increase in the transmittance from 47% to 85% at 600 nm (Supplementary Fig. 9). The excellent UV-blocking properties are ascribed to the existence of phenylpropane structures and phenolic hydroxyl groups in the lignin molecules with UV absorption ability. Consequently, the aesthetic wood treated for 2 h exhibited a good UV absorption performance at the range of 200–400 nm, a high average transparency (80%) at 600 nm, and a low reflectance at the visible wavelengths (Fig. 4b).

Subsequently, more types of patterns can be realized by stacking multiple layers of aesthetic wood. For example, various lattice patterns can be designed by stacking two layers of aesthetic wood rotated at an angle relative to each other. Based on the high transmittance and intrinsic aesthetic, this capability can enable the potential application on patterned ceilings (Fig. 4c). Moreover, the abundance of patterns can be further increased by developing aesthetic wood using other wood species (mainly softwoods) through this universal fabrication method. Here, another type of esthetic wood was also fabricated using pine (Supplementary Fig. 10). Double-layer pine aesthetic wood and one-layer pine aesthetic wood with one-layer Douglas fir aesthetic wood both show various aesthetic patterns (Supplementary Fig. 10b–d).

Furthermore, we evaluated the weathering stability of aesthetic wood-R and -L by exposing the materials outdoors for 3 weeks and measuring the optical properties, including the transmittance and haze (Supplementary Fig. 11a, b). Compared with the original aesthetic wood-R, the transmittance of the outdoor-exposed aesthetic wood-R decreased slightly while the haze increased from ~93% to ~98% in the wavelength range of 400–800 nm. Moreover, the same trends in the transmittance and haze occurred in esthetic wood-L as well. Additionally, we compared the mechanical properties between the samples before and after outdoor exposure (Supplementary Fig. 11c, d). There was no significant degradation in the strength of the aesthetic wood. These results indicate the aesthetic wood’s strong short-term weathering stability, which suggests the material’s potential for practical applications. However, there may be durability concerns for long-term outdoor operation, which requires further exploration in future studies.

### Light guiding and anti-glare effect of aesthetic wood

The aesthetic wood also demonstrates excellent optical management capability including the anti-glare effect and light guiding, which are of great significance for transparent ceiling applications. The esthetic wood largely scatters the forward light, leading to a high optical haze of ~93% (Supplementary Fig. 12a). SEM image of the fabricated aesthetic wood demonstrates inherited aligned microstructure (Supplementary Fig. 12b). With the refractive-index-matched polymer (e.g., epoxy) in wood lumina, light can propagate along the microchannels, which function as lossy waveguides^9^. Moreover, in order to demonstrate the optical management of the aesthetic wood used as pattern ceiling with high transparency and high haze, model houses designed with glass and aesthetic wood ceiling are compared in Supplementary Fig. 12c. The white light source created by a solar simulator was applied in this design model. In order to verify that the uniform indoor light distribution can be observed by using the aesthetic wood ceiling, we collected the light intensities of eight points in the designed house model via a calibrated Si detector from Thorlabs, respectively. As the results revealed in Supplementary Fig. 12d, in the glass model house, the maximum light intensity (56.8 mW cm^−2^) is about 17 times higher than the minimum light intensity (3.4 mW cm^−2^), leading to the non-uniform illumination. On the contrary, the diffused light distribution is much more uniform through the aesthetic wood ceiling because there is no obvious light intensity decrease between the brightest spot (48.2 mW cm^−2^) and the darkest spot (20.9 mW cm^−2^). Note that its high haze is the main reason, which changes the path of light propagation to avoid the appearance of strong glare as well^35^. Therefore, the aesthetic wood ceiling not only provides us a different experience of visual beauty and comfort but also enhances energy efficiency for indoor lighting owing to its high haze and anti-glare effects compared with a glass ceiling. Our aesthetic wood shows excellent performance in terms of its optical properties, mechanical strength, thermal insulation, UV-blocking, and aesthetic function, all of which make it stand out from previously reported transparent wood materials (Supplementary Table 2).

### Thermal insulation properties of aesthetic wood

**Fig. 5:**
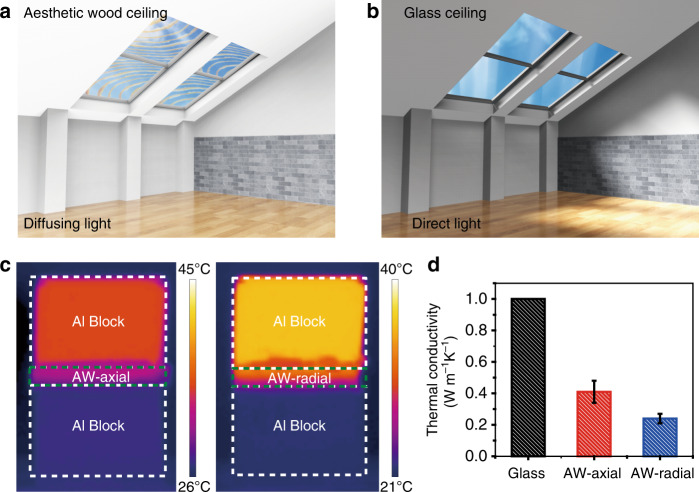
Light guiding effect and thermal insulation properties of aesthetic wood. **a**-**b** The schematic scene shows the light distribution and aesthetic appeal inside a building via applying the aesthetic wood (abbreviated as AW in the **d**) ceiling comparing with the glass ceiling. **c** IR images of aesthetic wood with temperature distributions in the axial (heat transfer direction is parallel to the aligned wood microchannels) and radial (heat transfer direction is perpendicular to the aligned wood microchannels) directions. **d** Thermal conductivities of glass^9^, axial and radial direction of our aesthetic wood (AW). Error bars represent standard deviation.

The aesthetic wood with good mechanical and optical performances can find potential applications as a patterned ceiling in a museum or gallery where its aesthetics can be showcased and can potentially replace glass ceiling (Fig. 5a, b). Simultaneously, aesthetic wood can also improve energy efficiency due to its excellent thermal insulation properties compared to glass^9,36^. As revealed in Fig. 5c, d, aesthetic wood exhibits a thermal conductivity of 0.24 W m^−1^ K^−1^ in the radial direction (perpendicular to the wood growth direction) which is a lower thermal conductivity than that in the axial direction. The heat transferred in the radial direction is restrained owing to the larger phonon scattering effect than in the axial direction (along the growth direction), which exhibits a thermal conductivity of around 0.41 W m^−1^ K^−1^^9^. For comparison, the isotropic thermal conductivity of common window glass is 1 W m^−1^ K^−1^, making aesthetic wood highly desirable from a thermal insulation perspective. The anisotropic thermal transport of aesthetic wood combined with low thermal conductivities is favorable for energy-efficient buildings. The superior thermal insulation to glass positions our developed esthetic wood to be a potential candidate for energy-efficient building materials.

To further illustrate the insulating effect of aesthetic wood ceilings applied in energy efficient buildings, we conducted a comparative evaluation in which a simplified house model for nighttime was used to calculate the indoor temperature change when single-pane glass ceilings are replaced with single-pane aesthetic wood ceilings. The house is assumed to have a floor area of 10 m × 10 m = 100 m^2^ and a 45° rooftop. The related parameters of the assumed house are described in Supplementary Table 3. The R-values and U-factors in Supplementary Table 3 are based on recommendations of the Department of Energy (https://www.homedepot.com/c/ab/insulation-r-valuechart/9ba683603be9fa5395fab9091a9131f, https://www.energy.gov/energysaver/design/windows-doors-and-skylights/doors)^37^. The total U-factors of the ceilings were calculated using the WINDOW 7.7 algorithm developed by Lawrence Berkeley National Laboratory (LBNL)^38^ (Supplementary Fig. 13a). Based on energy balance, the heat loss should be equal to the indoor heating power *P*_h_ when the indoor temperature *T*_in_ is higher than the outdoor temperature *T*_out_, thus we have equation:

1 $$(T_{{\mathrm{in}}} - T_{{\mathrm{out}}})\left(\mathop {\sum}\limits_{i = 1}^4 {\frac{{A_i}}{{R{\mathrm{ - value}}_i}}} + U_5A_5 + U_6A_6\right)\,{\mathrm{ = }}\,P_{\mathrm{h}}.$$

We can write it as

2 $$\Delta T\,{\mathrm{ = }}\,P_{\mathrm{h}}R,$$

where the temperature difference $\Delta T\,{\mathrm{ = }}\,T_{{\mathrm{in}}} - T_{{\mathrm{out}}}$ (°C) and the absolute thermal resistance $R\,{\mathrm{ = }}\frac{1}{{\mathop {\sum}\limits_{i = 1}^4 {\frac{{A_i}}{{R{\mathrm{ - value}}_i}}} + U_5A_5 + U_6A_6}}$ (°C W^−1^) When single-pane glass ceilings are replaced with single-pane aesthetic wood ceilings under the same heating power *P*_h_, *R* and Δ*T* will change accordingly. Supplementary Fig. 13b shows the relative change of Δ*T* (%) when single-pane glass ceilings are replaced with single-pane aesthetic wood ceilings with different thicknesses. If Δ*T* is 30 °C in a balanced state when glass ceilings are used, the change of indoor temperature (the outdoor temperature does not change) when aesthetic wood ceilings are used should be approximately +2.43 °C for the 6-mm-thick aesthetic wood-L and +0.81 °C for the 2 mm-thick aesthetic wood-L. In cases when the indoor temperature is lower than the outdoor temperature (summer), the indoor temperature would decrease in a similar manner. The above results indicate that the aesthetic wood with integrated advantageous mechanical, optical, thermal and aesthetic features holds promise for sustainable energy-efficient buildings^39^.

## Discussion

In this work, we demonstrated an aesthetic transparent wood with inherited wood patterns. Owing to the quite variable density, pore distribution, and lignin distribution in different wood tissues (e.g., EW, LW), selective delignification can be applied to natural wood for fabricating aesthetic wood with preserved growth ring patterns. Moreover, the efficient delignification process and remaining lignin successfully contribute to the large-scale fabrication of aesthetic wood. Consequently, the aesthetic wood with perpendicular lumina possesses a high transparency of ~80% with excellent UV-blocking properties and a high optical haze of ~93%, which leads to good anti-glare performance and light guiding to create a comfortable space contrasting with glass. The aesthetic wood also exhibits excellent mechanical properties including a high strength of 91.95 MPa, a high toughness of 2.73 MJ m^−3^ and a low thermal conductivity of 0.24 W m^−1^ K^−1^. All these characteristics position the aesthetic wood as a promising candidate for green building materials, energy-efficient buildings and other construction applications with added aesthetic functions, particularly as an attractive patterned ceiling.

## Methods

### Materials and chemicals

Douglas fir wood was purchased from Home Depot. Bass wood was purchased from Walnut Hollow Company. Balsa wood was purchased from Midwest Products Company. Pine wood was purchased from Amazon. The chemicals used for the delignification process were sodium chlorite (NaClO_2_, ~80%) and acetic acid purchased from Sigma-Aldrich. The solvents including deionized (DI) water and ethanol alcohol (95%, Pharmco-Aper) were used. The polymer Aero-Marine epoxy resin (#300 and #21) was used.

### Delignification process and polymer infiltration

The lignin removal solution was prepared by dissolving NaClO_2_ powder in DI water and adding acetic acid to adjust the pH value (~4.6). The wood samples were placed in the NaClO_2_ solution and kept boiling state for 2 h until the EW became white. Afterwards, the delignified wood samples were rinsed with DI water for three times and kept in ethanol. Subsequently, the as-prepared epoxy resin was used for thorough infiltrating into the delignified wood scaffold. The aesthetic wood was fabricated after a 24 h solidification.

### Characterizations

The morphologies of the wood samples were characterized by Tescan XEIA FEG SEM. The transmittance (*T*), haze and reflectance (*R*) were measured via the UV–vis Spectrometer Lambda 35 (PerkinElmer, USA) equipped with an integrating sphere. The absorbance spectra (*A*) was defined based on the transmittance (*T*) and reflectance (*R*) (*A* = 1 − *T* − *R*). The aesthetic wood ceiling and glass ceiling with a dimension of 60 mm × 60 mm × 2 mm are employed for the house models to test the light guiding effect. Thereinto, a Xenon lamp of the solar simulator from Newport was applied as the white light source with an illumination area of 5 cm in diameter. The samples surfaces were polished with a microtome (Leica, Germany) for Raman spectroscopy measurement, which was performed with a confocal Raman microscope (Renishaw inVia, Wotton-under-Edge, England) using a 785 nm laser and a water immersion objective (Nikon, 60×). The integration time 2 s and a step size of 600 nm were used in the measurement. Cosmic ray removal and baseline correction of the spectra were performed in the software Wire 3.2. For the VCA the mapping data were exported into CytoSpec (commercially available MatLab based software). The mechanical performance was assessed by a tensile tester (Instron) and three specimens (with a length of 90 mm and a width of 60 mm) were used to obtain the average values. The Steady State Laser-Infrared Camera Thermal Conductivity Characterization System^40^ was used to test the thermal conductivities. As demonstrated in Fig. 5, the sandwich structure where consisted of one sample (1 cm × 1 cm × 2 mm) in the middle of two Al blocks. The corresponding temperature contribution was recorded by the IR camera.

## Supplementary information

Supplementary Information

## Data Availability

The data that support the findings of this study are available from the corresponding author upon reasonable request.
