# Supplementary material for: Scalable aesthetic transparent wood for energy efficient buildings
Source: Nat Commun. 2020 Jul 31;11:3836. doi: 10.1038/s41467-020-17513-w (PMC7395769; doi:10.1038/s41467-020-17513-w)
Supplement: Supplementary file 1 — Supplementary Information [file 41467_2020_17513_MOESM1_ESM.pdf]

Supplementary Information

Scalable Aesthetic Transparent Wood for Energy Efficient Buildings

Mi et al.

Supplementary Figures:

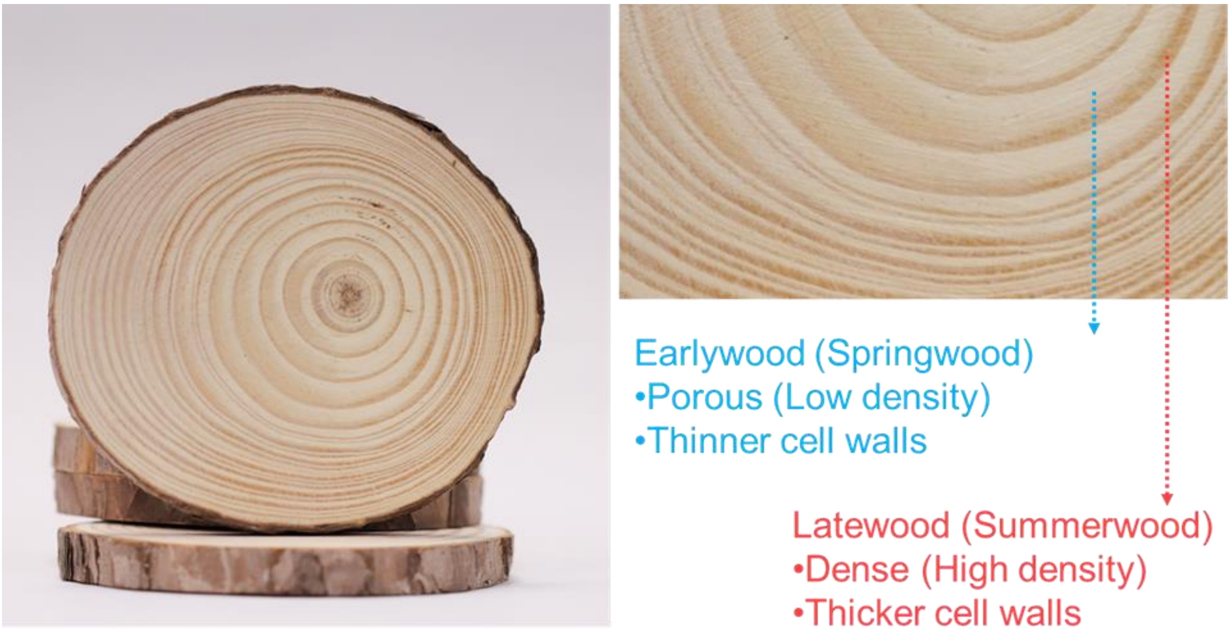

Supplementary Figure 1 | The macrostructural characteristics of the natural softwood. The photographs of natural softwood disk (Pine) with obvious annual growth rings: earlywood (Springwood) and latewood (Summerwood).

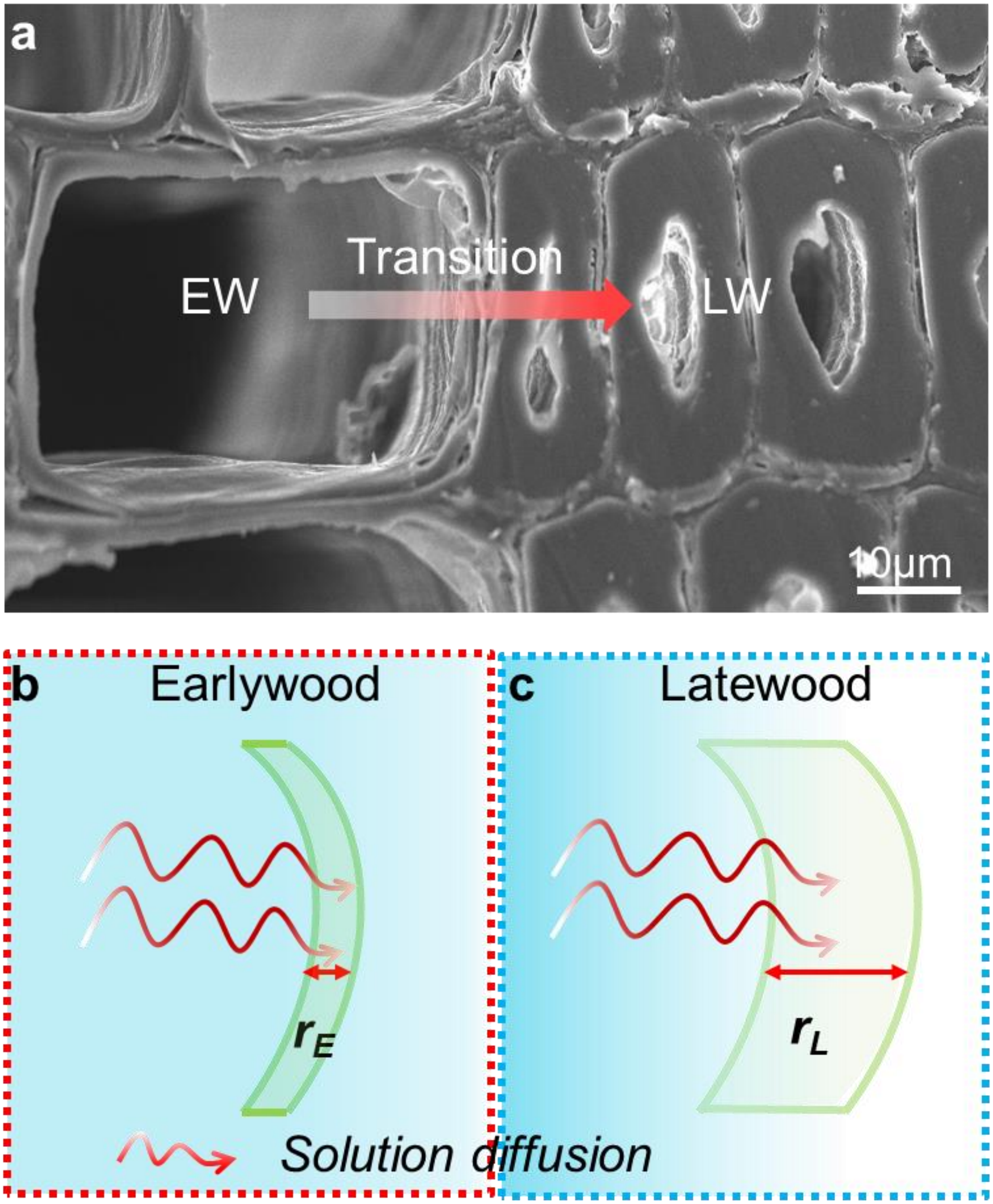

Supplementary Figure 2 | The solution diffusion processes in earlywood and latewood. (a) The SEM image of transition part from earlywood to latewood in cross-section of natural Douglas fir wood. (b)-(c) The solution diffusion processes in earlywood and latewood: the thinner cell wall (r_E) is prone to be fully infiltrated.

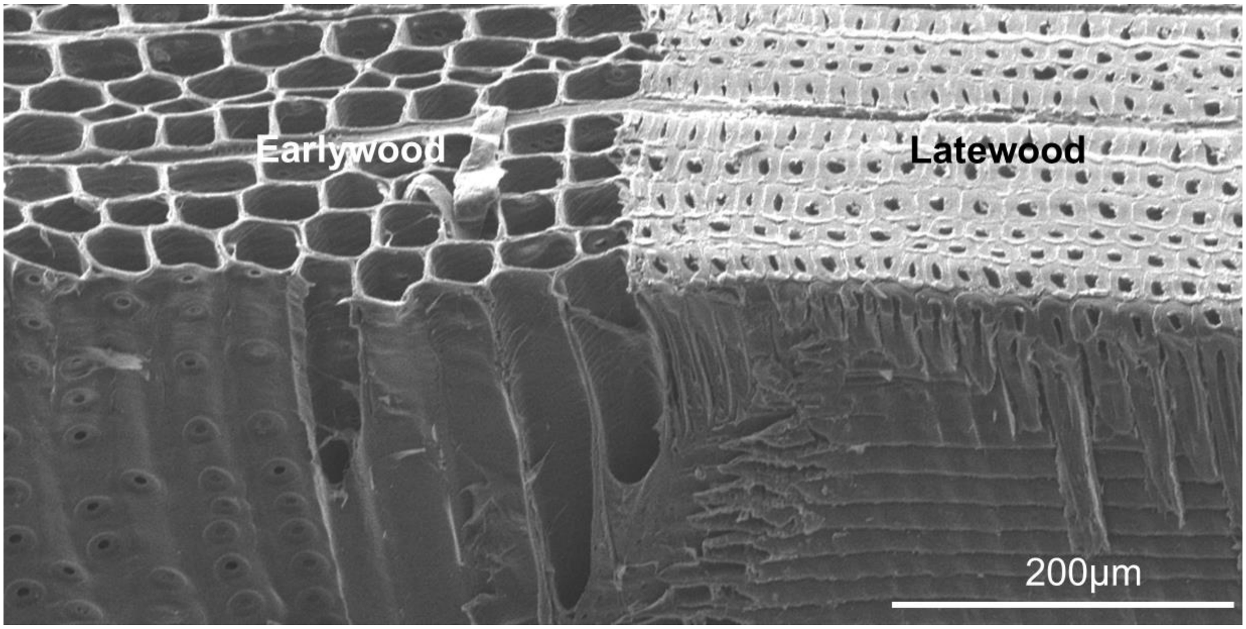

Supplementary Figure 3 | The SEM image showing the microstructure of the Douglas fir after spatially-selective delignification. The structure and aesthetic patterns are well-preserved after spatially-selective delignification, including the earlywood (left) with larger lumina, thinner cell walls than latewood (right).

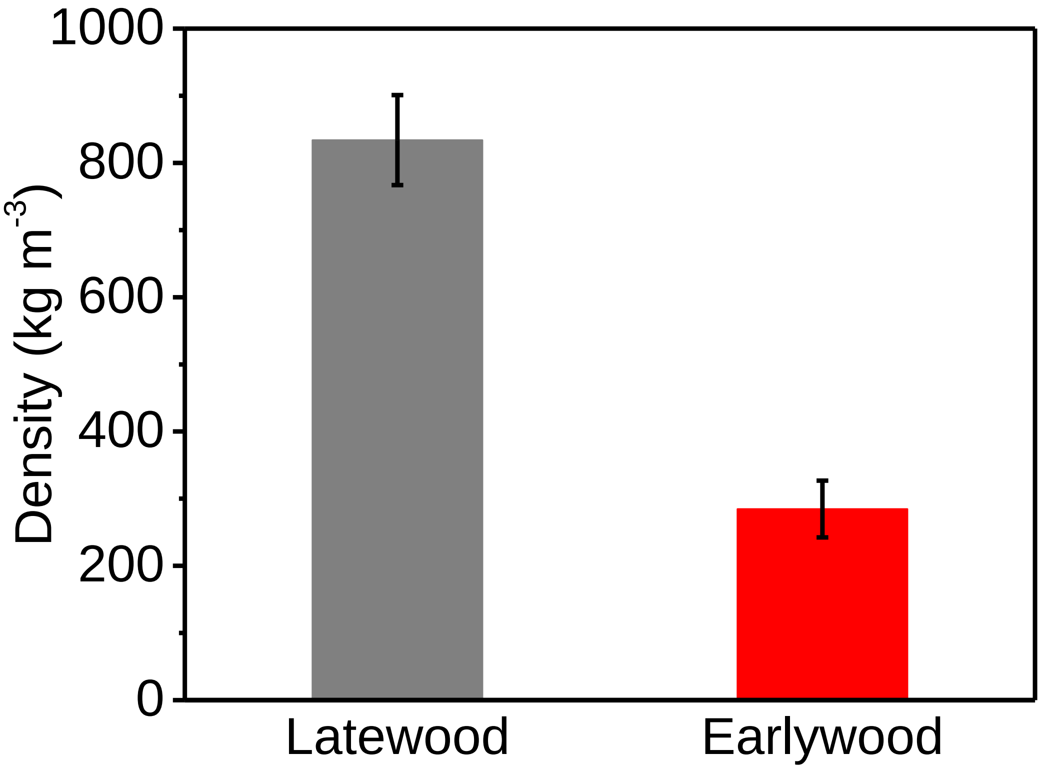

Supplementary Figure 4 | The density comparison between earlywood and latewood in Douglas fir. Latewood possesses much higher density than Earlywood.

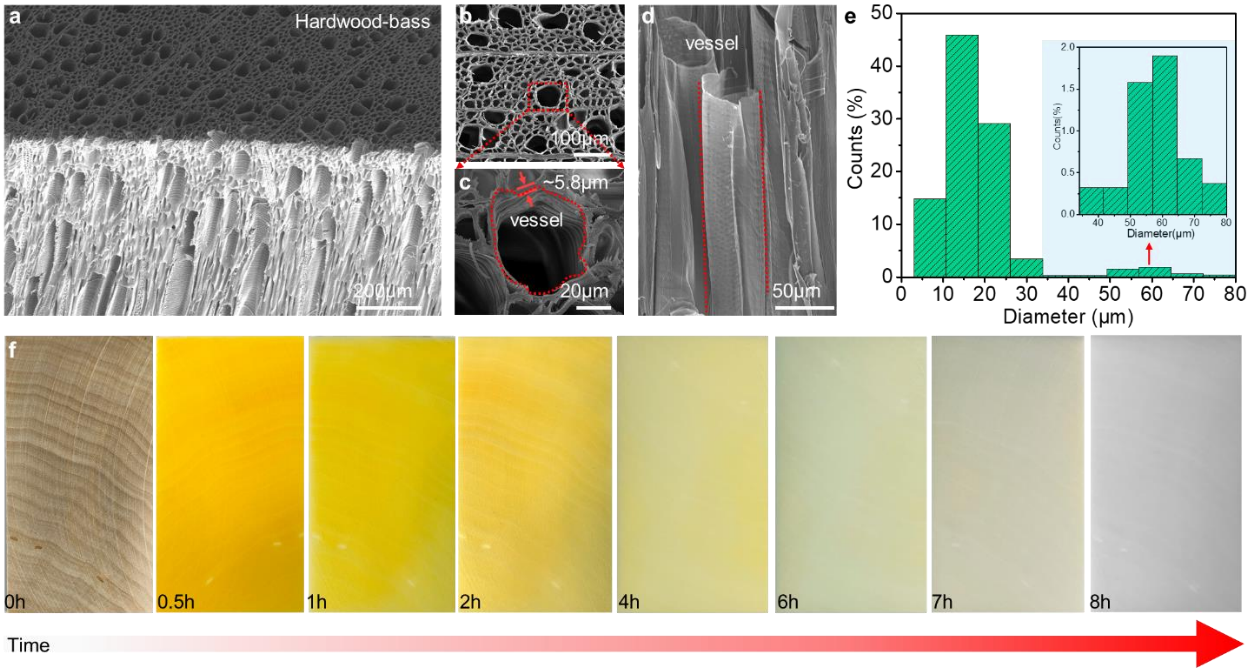

Supplementary Figure 5 | Morphological and chemical characterizations of basswood. (a) The SEM images of basswood showing its mesoporous structure. (b)-(c) The top view image of bass to show the lumen and vessel channels. (d) The vessel channel in the basswood. (e) The diameter distribution of fiber tracheids and vessels in the hardwood. (f) The photos of basswood templates during lignin removal process in the laboratory to show the color and pattern changes.

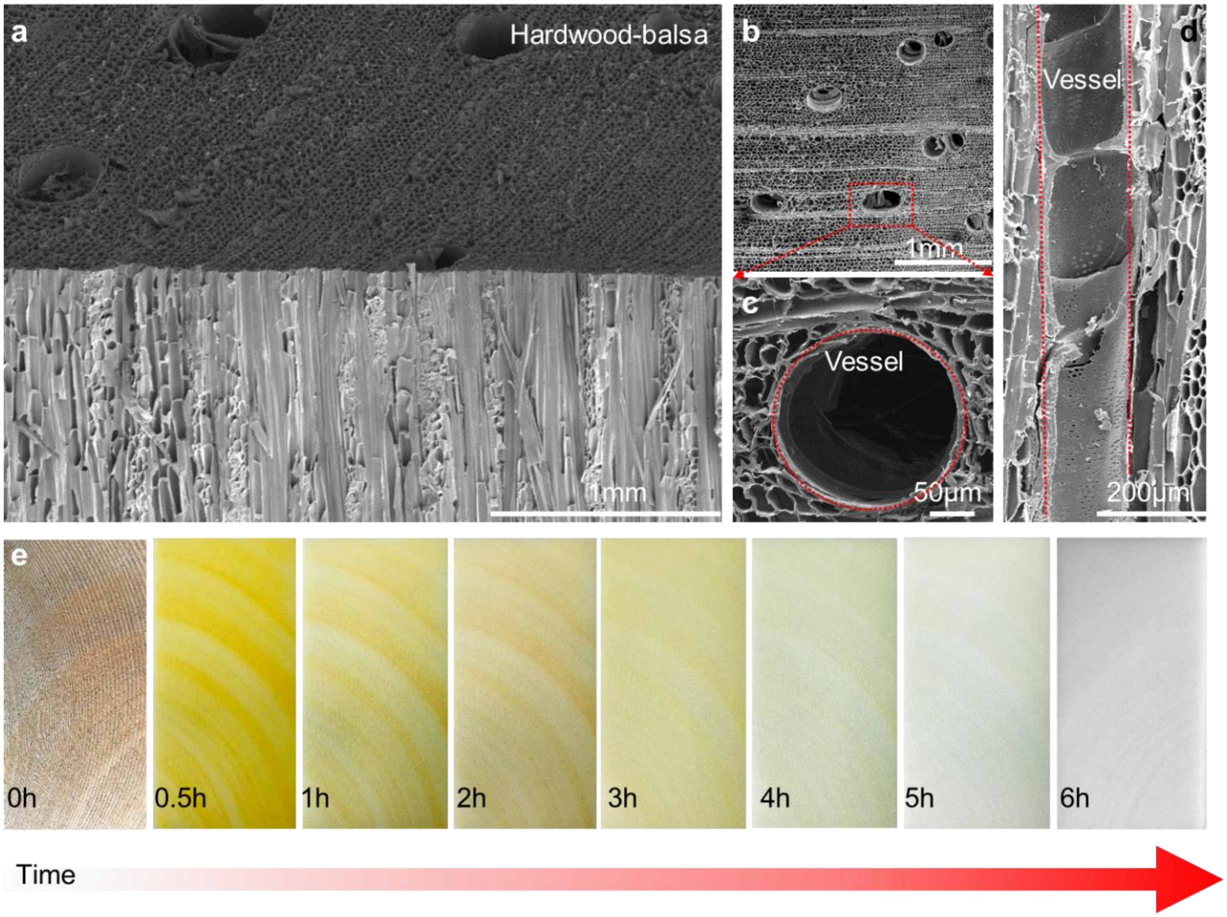

Supplementary Figure 6 | Morphological and chemical characterizations of balsa wood. (a) The SEM images of balsa showing its mesoporous structure. (b)-(c) The top view image of balsa to show the fibre lumina and vessel channels. (d) The vessel channel in the balsa wood. (e) The photos of balsa templates during lignin removal process in the laboratory to show the color and pattern changes.

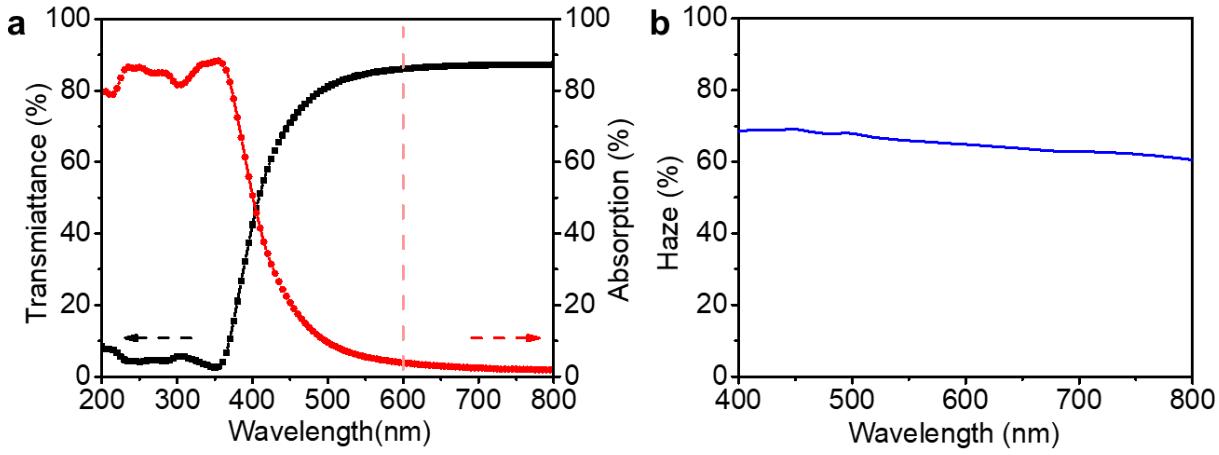

Supplementary Figure 7 | The optical properties of aesthetic wood-L. (a) The large-scale aesthetic wood-L (0.6 mm) exhibits high transmittance of 87% at 600 nm and strong absorption at the range of UV-spectrum; (b) The haze is measured as 65% at 600 nm.

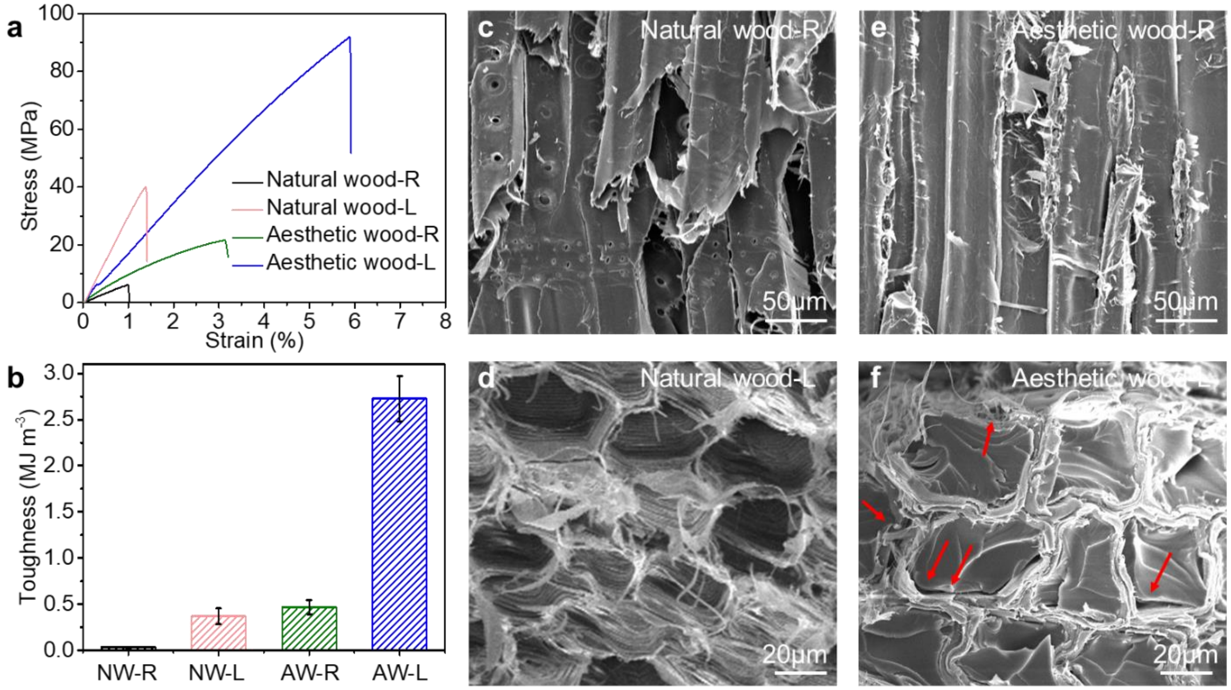

Supplementary Figure 8 | The mechanical properties of aesthetic wood. (a-b) The mechanical properties comparison of natural wood and aesthetic wood including stress-strain curves and toughness histogram. The aesthetic wood-L (AW-L) shows superior performance to aesthetic wood-R (AW-R). (c-d) SEM images of the fracture surface of the natural wood after tensile tests of two types. (e-f) SEM images of the fracture surface of the aesthetic wood after tensile test of two types.

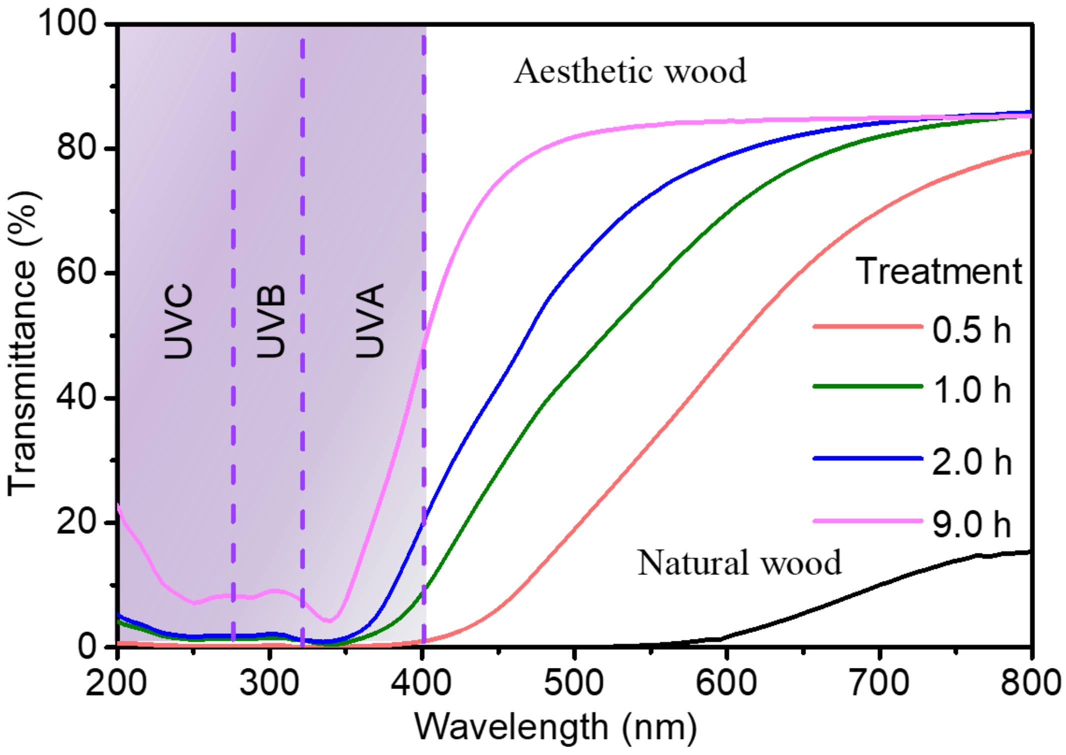

Supplementary Figure 9 | The tunable UV-blocking properties of aesthetic wood-R (2 mm-thick) prepared by different delignification treatment durations.

.

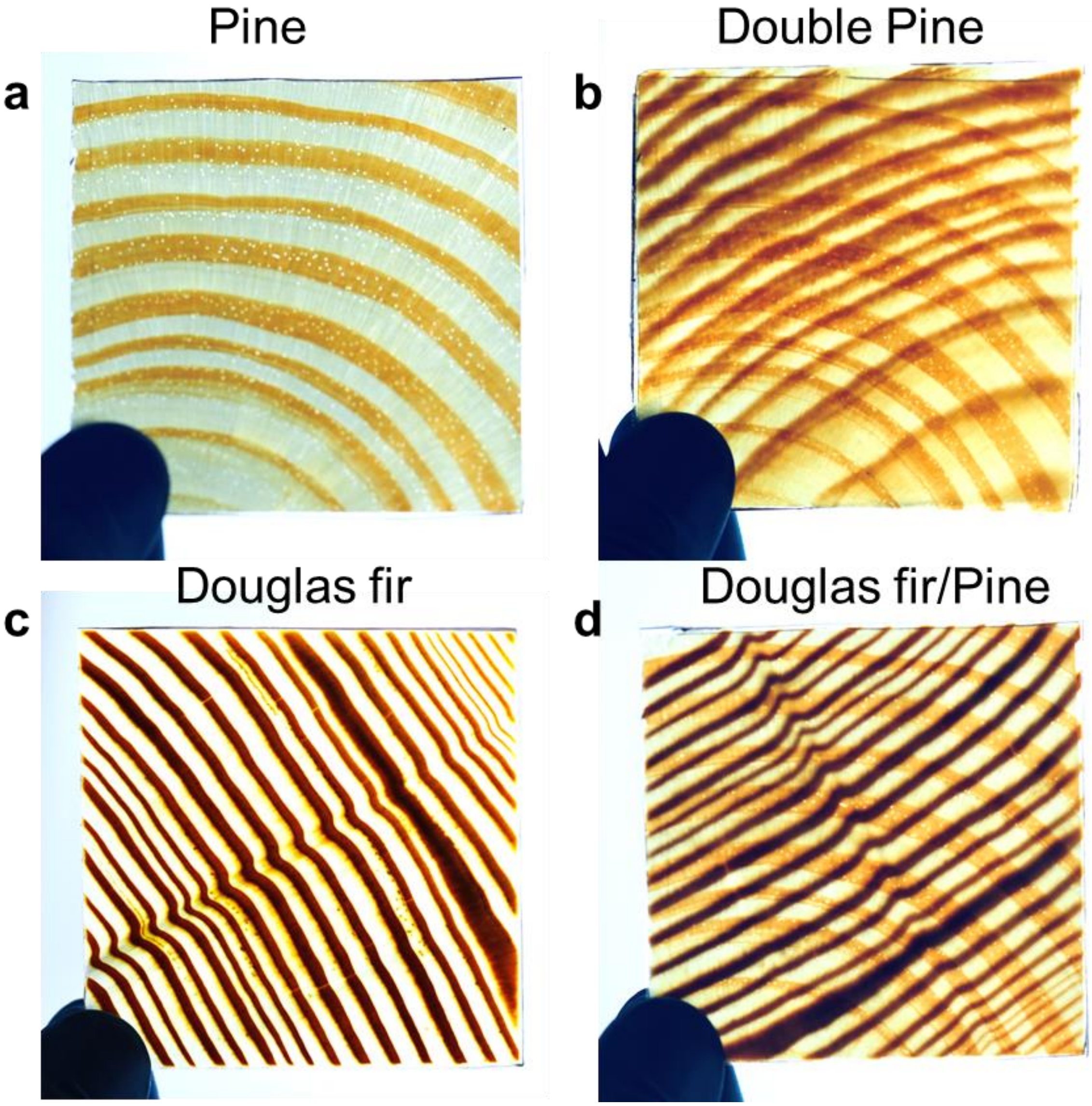

Supplementary Figure 10 | Aesthetic wood patterns achieved by stacking of aesthetic wood slices from the same or different wood species. (a) The aesthetic wood fabricated from pine wood. (b) An aesthetic wood pattern is formed by stacking two layers of pine wood. (c) The aesthetic wood fabricated from Douglas fir wood. (d) An aesthetic wood pattern is formed by stacking one layer of pine wood and one layer of Douglas fir wood, which has a stronger potential for aesthetic applications.

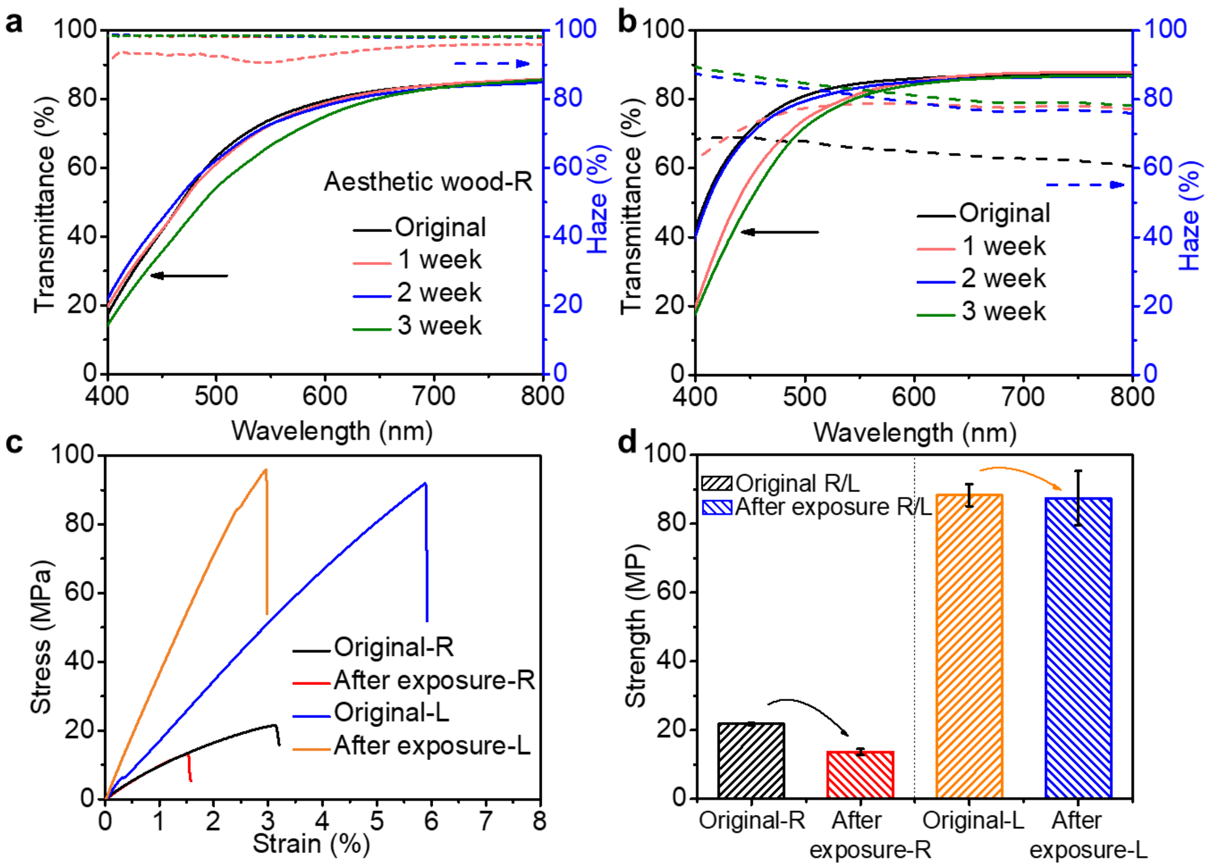

Supplementary Figure 11 | The evaluation of the weathering stability of aesthetic wood. The optical properties of the original (a) aesthetic wood-R (2 mm) and (b) aesthetic wood-L (0.6 mm) before and after exposure to sunlight for 1, 2, and 3 weeks. (c)-(d) Comparison of the tensile properties of the samples before and after 3 weeks of outdoor exposure.

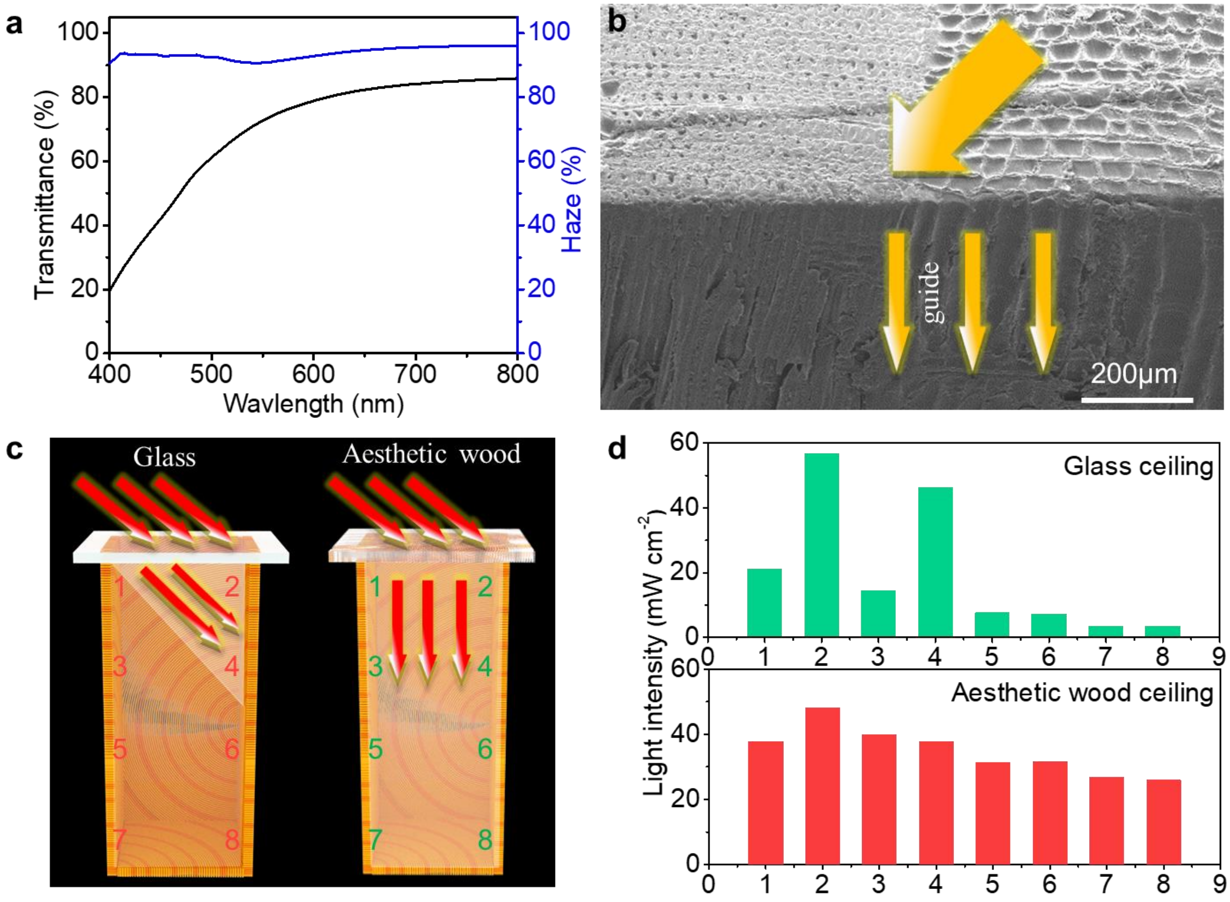

Supplementary Figure 12 | Light guiding and anti-glare effect of aesthetic wood. (a) A 2 mm-thick aesthetic wood ceiling exhibits good transparency and large scattering in the visible wavelength range with high optical haze. (b) An SEM image of the aesthetic wood structure, which maintains the aligned microchannels well so they can be used as micro-sized waveguides. (c) Schematics of the uniform light distribution inside the house model when using the aesthetic wood as a transparent ceiling in comparison to using glass. (d) The records for the light distribution at corresponding point locations in the aesthetic-wood-ceiling house and glass-ceiling house.

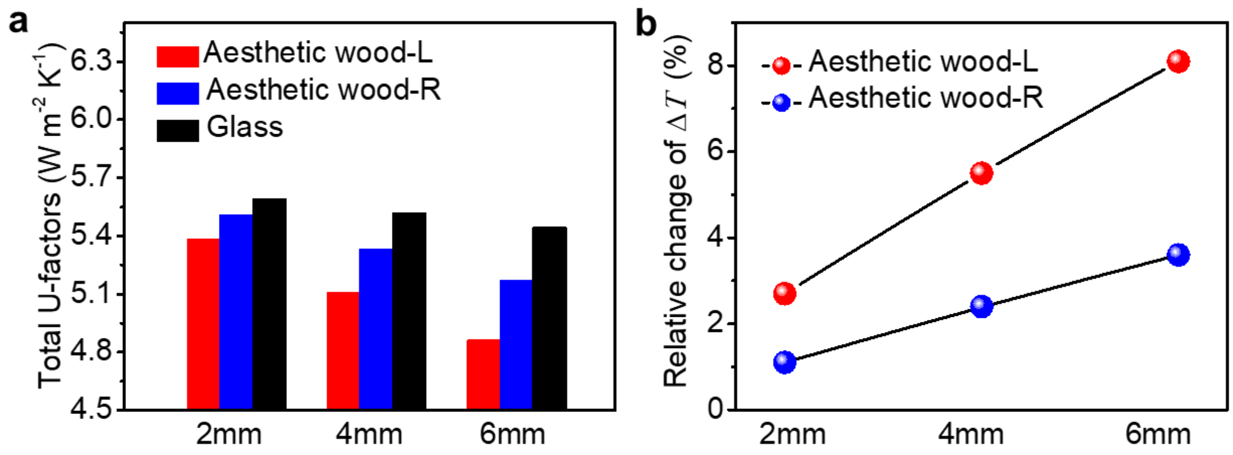

Supplementary Figure 13 | A comparative evaluation for thermal insulation properties of glass and aesthetic wood. (a) Total U-factors of the ceilings for different thicknesses of aesthetic wood and glass. (b) Relative change of ΔT (%) for different thicknesses of aesthetic wood when the glass ceilings were replaced by aesthetic wood ceilings.

Supplementary Tables:

Supplementary Table 1. Comparison of transparent wood sizes prepared in this study and reported in the literature. [a]

Reference	Wood species	Substrate	Polymer	Wood direction	L*W*T (mm^3)
**Our Work**	**Douglas fir**	**Intact wood**	**Epoxy**	**R**[b]	**86 x 86 x 2**
**Our Work**	**Douglas fir**	**Intact wood**	**Epoxy**	**L**[b]	**320 x 170 x 0.6**
Ref 1	Poplar	Wood fiber	PMMA	--	300 x 300 x 10
Ref 2	Bass	Intact wood	Epoxy	R	35 x 30 x 5
Ref 3	Bass	Intact wood	PVP	R	30 x 22 x 1
Ref 4	Balsa	Intact wood	PMMA	L	20 x 20 x 0.7
Ref 5	Beech	Intact wood	PMMA	R	60 x 30 x 5
Ref 6	Bass	Intact wood	PMMA	L	20 x 20 x 0.42
Ref 7	Poplar	Intact wood	PMMA	L	20 x 10 x 0.5

[a] Polymer: Infiltrating polymer; Wood direction: The wood direction of transparent wood, including R and L; Wood size: length (L)*width (W)*thickness(T) (mm^3)

[b] Wood direction (R): aligned micro-channels perpendicular to the wood plane;

Wood direction (L): channels parallel to the wood plane

Supplementary Table 2. The properties and performance of transparent wood prepared in this study and reported in the literature.

Reference	Transparent wood thickness (mm)	Wood direction	Transmittance (%)	UV-blocking	Tensile strength (MPa)	Thermal conductivity (W $m^{-1}K^{-1}$)	Aesthetic patterns
**Our work**	**2**	**R**	**80**	**Yes**	**21.56**	**0.41**	**Good**
**Our work**	**0.6**	**L**	**87**	**Yes**	**91.95**	**0.24**	**Good**
Ref 2	5	R	90	--	13	0.32	No
Ref 3	1	R	90	--	--	--	No
Ref 4	0.7	L	91.7	--	90.1	--	No
Ref 5	5	R	86	--	59.8	--	No
Ref 6	0.42	L	61	--	171.4	--	No
Ref 7	0.5	L	86.7	--	45.92	--	No
Ref 8	1.5	R	83	--	100.7	0.23	No

Supplementary Table 3. Parameters of the house model.

Number	Surface	Area (m^2)	Total U-factor ($W\ m^{-2}\ K^{-1}$)	R-value ($m^2\ K\ W^{-1}$)
1	Wall	182		15
2	floor	100		30
3	Rooftop	129.88		50
4	Door	3		5
5	Ceiling	1.2×1.2×8		
6	Window	25	0.3	

Supplementary references:

1. Wang, X. *et al.* Large-Size Transparent Wood for Energy-Saving Building Applications. *ChemSusChem* **11**, 4086-4093 (2018).
2. Li, T. *et al.* Wood Composite as an Energy Efficient Building Material: Guided Sunlight Transmittance and Effective Thermal Insulation. *Advanced Energy Materials* **6**, 1601122 (2016).
3. Zhu, M. *et al.* Transparent and haze wood composites for highly efficient broadband light management in solar cells. *Nano Energy* **26**, 332-339 (2016).
4. Li, Y., Fu, Q., Yu, S., Yan, M. & Berglund, L. Optically Transparent Wood from a Nanoporous Cellulosic Template: Combining Functional and Structural Performance. *Biomacromolecules* **17**, 1358-1364 (2016).
5. Yu, Z. *et al.* Transparent wood containing CsxWO3 nanoparticles for heat-shielding window applications. Journal of Materials Chemistry A 5, 6019-6024 (2017).
6. Wu, J. *et al.* Impact of delignification on morphological, optical and mechanical properties of transparent wood. *Composites Part A: Applied Science and Manufacturing* **117**, 324-331 (2019).
7. Gan, W. *et al.* Luminescent and Transparent Wood Composites Fabricated by Poly(methyl methacrylate) and γ-Fe_2O_3@$YVO_4:Eu^{3+}$ Nanoparticle Impregnation. *ACS Sustainable Chemistry & Engineering* **5**, 3855-3862 (2017).
8. Li, Y. *et al.* Lignin-Retaining Transparent Wood. *ChemSusChem* **10**, 3445-3451 (2017).
